# Chemical Composition and Evaluation of Insecticidal Activity of *Calendula incana* subsp. *maritima* and *Laserpitium siler* subsp. *siculum* Essential Oils against Stored Products Pests

**DOI:** 10.3390/molecules27030588

**Published:** 2022-01-18

**Authors:** Sara Basile, Natale Badalamenti, Ornella Riccobono, Salvatore Guarino, Vincenzo Ilardi, Maurizio Bruno, Ezio Peri

**Affiliations:** 1 Faculty of Forestry and Wood Sciences, Czech University of Life Sciences Prague, CULS, Kamýcká 129, 16500 Prague, Czech Republic; basile@fld.czu.cz; 2 Department of Agricultural, Food and Forest Sciences (SAAF), University of Palermo, Viale delle Scienze, Building 5, 90128 Palermo, Italy; ornella.riccobono94@gmail.com (O.R.); ezio.peri@unipa.it (E.P.); 3 Department of Biological, Chemical and Pharmaceutical Sciences and Technologies (STEBICEF), Università di Palermo, Viale delle Scienze, 90128 Palermo, Italy; vincenzo.ilardi@unipa.it (V.I.); maurizio.bruno@unipa.it (M.B.); 4 Institute of Biosciences and Bioresources (IBBR), National Research Council of Italy (CNR), Corso Calatafimi 414, 90129 Palermo, Italy; 5 Centro Interdipartimentale di Ricerca “Riutilizzo Bio-Based Degli Scarti da Matrici Agroalimentari” (RIVIVE), Università di Palermo, 90128 Palermo, Italy; 6 Interuniversity Center for Studies on Bioinspired Agro-Environmental Technology (BAT Center), University of Napoli Federico II, 80055 Portici, Italy

**Keywords:** GC×GC-MS analysis, cubebene derivatives, *Necrobia rufipes*, *Sitophilus oryzae*, *Lasioderma serricorne*, *Rhyzopertha dominica*

## Abstract

The problems of the environment and human health related to the use of synthetic and broad-spectrum insecticides have increasingly motivated scientific research on different alternatives and among these, the use of green systems, such as essential oils, have been explored. Several species of the Apiaceae and Asteraceae families, aromatic herbs rich in secondary bioactive metabolites, are used in the industrial field for pharmaceutical, cosmetic, and food purposes. Different essential oils extracted from some species of these families have shown acute toxicity and attractive and/or repellent effects towards different insects. In our work, we investigated the toxic potential of *Calendula incana* subsp. *maritima* and *Laserpitium siler* subsp. *siculum* essential oils against four insect species, *Sitophilus oryzae*, *Lasioderma serricorne*, *Necrobia rufipes*, and *Rhyzoperta dominica,* which are common pests of stored products. The composition of both oils, extracted by hydrodistillation from the aerial parts of the two plants, was evaluated by GC×GC-MS. *Calendula incana* subsp. *maritima* essential oil was rich in oxygenated sesquiterpenoids, such as cubebol (35.39%), 4-*epi*-cubebol (22.99%), and cubenol (12.77%), while the *Laserpitium siler* subsp. *siculum* essential oil was composed mainly of monoterpene hydrocarbons, such as *β*-phellandrene (42.16%), limonene (23.87%), and *β*-terpinene (11.80%). The toxicity Petri dish bioassays indicated that *C. maritima* oil killed a mean of 65.50% of *S. oryzae* and 44.00% of *R. dominica* adults, indicating a higher biocidal activity in comparison with *L. siculum* oil, while toward the other species, no significant differences in mortality were recorded. *Calendula maritima* oil could be, then, considered a promising candidate for further tests as an alternative biocide toward *S. oryzae* and *R. dominica*. The possibility that the relatively high content of oxygenated sesquiterpenoids in *C. maritima* essential oil determines its higher biocidal activity is discussed.

## 1. Introduction

Food spoilage during storage determined by pest infestation is a major concern in both developed and developing countries, causing from 9 to 20% of product losses and consequent strong economic impact in terms of quality and market access [1,2]. Stored food products are reported to potentially be affected by about 1660 insect species worldwide [3], the most dangerous of them mainly belonging to only three orders, Coleoptera (beetles), Lepidoptera (moths), and Psocoptera (psocids) [4]. In the past, stored product pests have been mainly controlled by synthetic contact pesticides [5]. However, this approach determined increasing resistance phenomena [6] and had a negative impact on the environment and human health [7,8].

The drawbacks of the widespread use of chemical insecticides at ecotoxicological, environmental, and social levels have led researchers to find suitable alternatives that are more environmentally friendly than synthetic chemicals in the management of stored product pests [9]. Recently, the guidelines of the European Union have been aimed at a reduction in the use of synthetic chemicals in favour of other eco-friendlier approaches based on natural products [10]. In this context, the use of insecticides based on plant extracts is attracting extensive attention both among researchers and consumers [9]. In detail, academic interest in plant natural products with insecticidal properties has continued to develop in the past 20 years, especially in countries such as China, Iran, Turkey, and India, where regulation on the use of such botanicals have more relaxed regulatory requirements in comparison with the EU and the USA [11].

Nevertheless, despite this growing interest, many valuable plants and their metabolites have not yet been explored; therefore, it is essential to conduct new studies on various wild species to evaluate their deterrent and insecticidal properties [12].

In this regard, the use of essential oils (EOs) as natural insecticides is growing enormously, thanks to their high biodegradability and wide bioactivities [13,14]. The metabolites present in the different EOs possess multiple properties due to single action or synergistic action, such as acute toxicity, feeding and oviposition deterrence, repellence, and attraction [15,16]. Although the mechanisms of action are not perfectly known, several studies have shown that the greatest toxicity is caused by the interaction of the oil with the nervous system of insects mediated by the inhibition of acetylcholinesterase (AChE), or by the antagonism of octopamine receptors [17].

In this study, we investigated the EOs of two punctiform species belonging to the genus *Calendula* and *Laserpitium* growing in Sicily. The genus *Calendula* (Asteraceae) is distributed in all of Europe and North Africa [18]. *Calendula incana* subsp. *maritima* (Guss.) Ohle [19] [Syn. *Calendula maritima* Guss.; *Calendula suffruticosa* subsp. *maritima* (Guss.) Meikle], hereafter named *C. maritima*, is a perennial and suffruticose plant, 20–40 cm tall, with branched and creeping stems. The leaves, rounded at the apex, fleshy, have a pungent odor and a length of 20–45 mm. Inflorescences in flower heads of 3–5 cm in diameter, with yellow female flowers.

*Laserpitium siler* subsp. *siculum* (Spreng.) Santangelo, F. Conti & Gubellini [20] [Syn. *Laserpitium siculum* Spreng.; *L. garganicum* (Ten.) Bertol.; *Siler siculum* Thell.; *L. garganicum* (Ten.) Bertol. subsp. *siculum* (Spreng.) Pignatti; *Siler montanum* Crantz subsp. *siculum* (Spreng.) Iamonico, Bartolucci et F. Conti], hereafter named *L. siculum*, instead, is a herbaceous plant 2–5 dm tall, hairless, and glaucescent. The stem is erect, streaked, with basal leaves of 1–2.5(4) dm. The umbels have 10–20 rays, and the fruit has 1.5 mm wide wings. It blooms between June and July. *Laserpitium siculum* is distributed over an area that covers the central-southern Apennine regions, Sicily, and the Dinaric Alps [21], and it is distinguished from *L. garganicum* (Ten.) Bertol. (endemism of central-southern Italy and Sardinia) above all for the shape and length of the bracts.

*Calendula maritima* and *L. siculum* are species that are growing in marginal lands, and their cultivation could be exploited in such contexts where other cultivated species cannot be planted. Furthermore, given the excellent results shown by the EOs obtained from different species of the Umbelliferae family [22], used as natural insecticides [23,24], and considering the insecticidal and repellent properties of the *Calendula* genus [25,26], the object of this study was to investigate, for the first time, the chemical composition of *C. incana* subsp. *maritima* and *L. siler* subsp. *siculum* EOs, and to evaluate their biocidal effect on four species of stored products pests: *Sitophilus oryzae* L. (Coleoptera: Curculionidae), *Lasioderma serricorne* F. (Coleoptera: Anobiidae), *Necrobia rufipes* DeGeer (Coleoptera: Cleridae), and *Rhyzoperta dominica* F. (Coleoptera: Bostrichidae). These species have a worldwide distribution range and are reported as among the most dangerous for stored commodities [27,28,29,30,31]. *Sitophilus oryzae*, also known as the rice weevil, and *R. dominica*, also named as the lesser grain borer, are primary insects of cereal grains in storage, including wheat, maize, and rice [32,33]. *Lasioderma serricorne*, the cigarette beetle, is a cosmopolitan polyphagous pest dangerous in the food and tobacco industry in many regions of the world [34]. *Necrobia rufipes*, commonly named the red-legged ham beetle, is a pest of stored products of animal origin, and its infestations are an increasing problem for the pet food industry [35]. The economic damage determined by these species is both quantitative due to substrate weight loss caused by insect feeding and qualitative due to product alterations such as loss of nutritional and aesthetic value, increased levels of rejects in the grain mass, and loss of industrial characteristics [36,37].

## 2. Results and Discussions

### 2.1. Chemical Composition of Essential Oil

Hydrodistillation of *C. maritima* aerial parts gave a yellow, dense oil. Overall, twenty-four compounds were identified, representing 95.78% of the total components, listed in Table 1 according to their retention indices on a DB-5 MS column and classified into two main classes based on their chemical structures. Oxygenated sesquiterpenes formed the main class, representing 87.77% of the oil, with cubebol (35.39%) as the most abundant component. In the same class, 4-epi-cubebol (22.99%), *τ*-muurolol (13.11%), and cubenol (12.77%) were also present. Sesquiterpene hydrocarbons was the other most abundant class (7.84%), with *α*-cubebene (1.12%) and *β*-cubebene (1.12%) as the main component of this class. Finally, other compounds such as monoterpenes were present in trace amounts.

From a comparison of the chemical compositions of EOs of other species belonging to the *Calendula* genus studied so far, it emerged that cubebane sesquiterpenes, such as *α*-cubebene, *β*-cubebene, 4-epi-cubebol, cubebol, and cubenol, the main compounds of our oil (60.62%), were present in a moderate amount only in some accessions of *C. arvensis* collected in Corsica [38] and *C. officinalis* collected in Turkey [39].

Among the other metabolites present in *C. maritima*, it is worth mentioning that τ-muurolol occurred in some accessions of *C. arvensis* collected in Corsica [38] and in populations of *C. officinalis* from Estonia [40] and South Africa [41], and cubenol derivatives were present in *C. arvensis* from Corsica [38] and *C. officinalis* from France [42]. Only two species, *C. arvensis* L. collected in Turkey [43] and *C. micrantha* studied in Egypt [44], did not show either cubebane derivatives or sesquiterpenes with the typical cadinene skeleton. All the other EOs extracted from *C. arvenis* L. [45,46,47,48,49], on the other hand, were characterized by a large presence of bicyclic sesquiterpenes, with the so-called cadalane (4-isopropyl-1,6-dimethyldecahydronaphthalene) carbon skeleton.

Hydrodistillation of *L. siculum* aerial parts gave light-blue oil. Overall, thirty-eight compounds were identified, representing 99.24% of total components, listed in Table 2 according to their retention indices on a DB-5 MS column and classified into five classes based on their chemical structures. The main class was constituted by monoterpene hydrocarbons, representing 90.38% of the oil, with *β*-phellandrene (42.16%), limonene (23.87%), and *β*-terpinene (11.80%) as the most abundant components. Oxygenated monoterpenes were the second most abundant class (5.47%), with 4-terpinenyl acetate (5.20%) as the main component of this class. On the other hand, the other chemical classes were present in traces. Despite the blue color of the oil, the presence of chamazulene was just 0.10%.

The presence of *β*-phellandrene, the main component of *L. siculum*, has been reported, only in moderate amount, in *L. garganicum* (14.4%) from Italy [50], *L. latifolium* from Stara Planina, Serbia (3.2%) [51] and Mt. Gučevo, Serbia (11.8%) [52], *L. pseudomeum* from Greece (6.7%) [53], and *L. zernyi* from Macedonia and Serbia (12.0–4.3%) [54]. Although several other EOs of previously studied taxa of *Laserpitium* showed to be particularly rich in monoterpene hydrocarbons, this compound was absent in *L. carduchorum* [55], *L. latifolium* [51,52,56], *L. petrophilum* [57], and *L. pseudomeum* [53]. It is interesting to point out that the only other accession of *L. siculum* studied so far, from plants collected in Central Italy [58], showed a completely different composition of the EO in comparison with our results, with a presence of monoterpene hydrocarbons ranging from 40.0 to 31.5% and abundant sesquiterpenes ranging from 39.3 to 20.4%. In this case, chamazulene occurred in quite a high amount (32.2–17.0%).

### 2.2. Toxicity Bioassays

**Figure 1:**
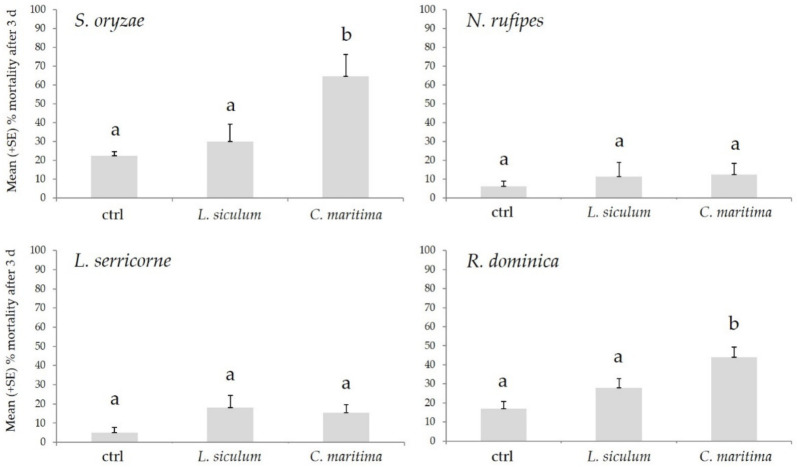
Mean (+SE) % adult mortality after three days (d) from treatment determined by *Calendula incana* subsp. *maritima* and *Laserpitium siler* subsp. *siculum* toward *Sitophilus oryzae*, *Necrobia rufipes*, *Lasioderma serricorne*, and *Rhyzoperta dominica.* Different letters indicate statistically significant differences (*p* < 0.05) among the treatments (one-way ANOVA, followed by Tukey’s test).

The biocidal effect of *C. maritima* and *L. siculum* EOs on *S. oryzae*, *R. dominica*, *N. rufipes*, and *L. serricorne* is reported in Figure 1. On *S. oryzae*, the EOs tested determined a different level of mortality (F_2,27_ = 6.63; *p* < 0.01; ANOVA), with the *C. maritima* EO that determined a 64.50% of dead adults, a value higher than using solvent (*p* < 0.001) or *L. siculum* EO (*p* < 0.05), while no statistical differences were recorded in the adult mortality between *L. siculum* and the solvent (*p* = ns). Similarly, the use of EOs toward *R. dominica*, determined differences statistically different in adult mortality (F_2.27_ = 8.56; *p* = 0.001; ANOVA), with *C. maritima* EO that determined a 44.00% of dead adults, a value higher than using solvent (*p* < 0.001) or *L. siculum* EO (*p* < 0.05), while no statistical differences were recorded in the adult mortality between *L. siculum* and the solvent (*p* = ns). Differently, the EOs tested didn’t determine differences in mortality levels toward *N. rufipes* (F_2.27_ = 0.72; *p* = ns; ANOVA) and *L. serricorne* (F_2.27_ = 2.12; *p* = ns; ANOVA), in comparison with the solvent.

Overall, these results indicate a clear toxicity effect of *C. maritima* EO exerted on *S. oryzae* and *R. dominica*. The deterrent effect toward insects of extracts obtained from plants belonging to the Calendula genus has already been reported in a few studies, but this is the first time it is observed from *C. maritima*. For example, Tavassoli and coworkers [25] observed that *C. officinalis* EOs has a repellent effect on the mosquito *Anopheles stephensi* Liston, similar to DEET, the most common repellent used worldwide. In another study, a *C. officinalis* extract was effective in reducing infestation of brassica pest insects such as the aphid *Brevicoryne brassicae* L., the beetles *Phyllotreta atra* F. and *P. nemorum* L., and the lepidopterans *Pieris rapae* L. and *Mamestra brassicae* L. [59]. Furthermore, a *C. arvensis* extract also determined toxic effects toward a few stored pest beetles, such as *Callosobruchus analis* Pic and *Tribolium castaneum* (Herbst) [26].

It is likely that the effect of *C. maritima* EO on the physiology of the tested insect is mainly related to the alteration of processes in the nervous system, such as the inhibition of acetylcholinesterase and positive allosteric modulation of GABA receptors that modify the neuronal activity, which, in turn, produce malfunctioning of nerve cells, as observed in other insects and mites [60,61,62]. However, in other cases, it has also been documented that EOs are modulators of the octopamine and tyramine receptors [63]. Furthermore, some EOs or their components can produce lipid peroxidation, increase the ionic permeability of the cell membrane, affect the integrity of DNA, and alter mitochondrial respiration [64,65].

The results obtained in our study, which indicate a strong biological activity toward *S. oryzae* and *R. dominica*, are probably determined by the high percentage of sesquiterpenes present in the *C. maritima* EO. Few studies suggest that sesquiterpenes present in plant leaves provide their primary chemical defenses against pathogens and insects by discouraging the herbivores activity by deterring substances [66].

In this context, the relevant amount of cubebol present in *C. maritima* EO could play an important role in deterring pests due to its potential toxic activity already reported in the literature of other EOs rich in this chemical [67,68]. Furthermore, the bioactivity of cubebol toward other insect species was suggested by testing the EOs of the leaves of *Psidium littorale* Raddi (Myrtaceae), rich in this sesquiterpene, against the larvae of *Anopheles gambiae* Giles (Diptera: Culicidae), evidencing high toxicity [68]. Additionally, Gu and coworkers [67] highlighted the role played by this molecule in its pure state when evaluating the insecticidal activity of the ethanolic extract of *Cryptomeria japonica* (D. Don) on larvae of the mosquitoes *Aedes albopictus* Skuse and *Aedes aegypti* L. Among the other main compounds present in *C. maritima* EO, cubenol can also have primary importance in determining the results obtained. In fact, this oxygenated sesquiterpene is highly present in the EO of *Pilgerodendron uviferum* (D. Don) Florin (Cupressaceae) that evidenced antifeedant effect toward *Hylastinus obscurus* Marsham (Coleoptera: Curculionidae) [69]. Similarly, muurolol, representing 13.11% of the total amount of *C. maritima* EO, was also one of the main components of *Zanthoxylum dissitum* Hemsl leaves and root extract, exhibiting toxicity toward the beetles *L. serricorne*, *T. castaneum*, and *Attagenus piceus* Olivier [70]. Among the other molecules present in this EO, *β*-cubenene has been found in echinacea oil, which has demonstrated strong insecticidal activity toward *S. granarius* and *T. castaneum* [71].

So far, no studies have been reported in the literature about the test of such molecules used singly or combined, and this can be the object of future experiments toward these species.

Concerning the *L. siculum* EO, our study did not evidence insecticidal activity toward the species tested even if the main monoterpenes present in the oils, such as limonene or phellandrene, have been reported to be toxic for stored product species. In fact, other EOs such as those extracted from *Pistacia lentiscus* L. and *Mentha haplocalyx* L., rich in these two monoterpenes, evidenced contact or fumigant toxicity toward *L. serricorne* [70,72].

In conclusion of our work, the experimental data obtained showed that the EOs tested exhibited promising bioactivity, in particular against *S. oryzae*. As we tested the whole EO and not its components singly, it is not possible to establish if the bioactivity was determined by a particular chemical or by the synergistic effect of the blend. However, in consideration of the data obtained in this study, the *C. maritima* EO and its main chemical constituents can be considered a promising candidate for further experiments in the laboratory and semi-field studies, in order to evaluate the LD_50_, the repellent properties, and the ability of such oils to protect the commodities from infestation by the stored pest.

## 3. Materials and Methods

### 3.1. Plant Materials and Essential Oils Extraction

The whole aerial parts of *C. maritima* were collected from the Trapani (Sicily) seafront near the Tonnara Tipa (38°01′58.29″ N; 12°31′39.60″ E), in May 2021. A voucher of the population analyzed was deposited in the herbarium of the University of Palermo (Voucher no. 109721). On the other hand, *L. siler* subsp. *siculum* aerial parts were sampled on Madonie Mountains (Sicily), on dolomitic slopes near Contrada Quacella (37°50′44.94″ N; 14°01′12.73″ E) 1500 m above sea level, in May 2020. A voucher of the population analysed was deposited in the herbarium of the University of Palermo (Voucher no. 109716).

A variable quantity of the dried aerial parts of *L. siculum* and *C. maritima* (155.00 and 1128.00 g, respectively) were ground and subjected to hydrodistillation for a period of 3 h using Clevenger’s apparatus (European Pharmacopoeia, 2020). The oils (yields 0.63% (*v*/*w*) and 0.09% (*v*/*w*), respectively) were dried with anhydrous sodium sulphate, filtered, and stored in the freezer at −20 °C, until the time of analysis.

### 3.2. GC×GC-MS Analyses

One μL of diluted EOs (2/100 *v*/*v*, in *n*-hexane) was injected into an Agilent 7000C GC (Agilent, Santa Clara, CA, USA) system equipped with a split/splitless injector and a GERSTEL automatic sampler (GERSTEL, Linthicum, MD, USA). The first column set was composed of a fused silica Agilent DB-5 MS capillary column (30 m × 0.25 mm I.D.; 0.25 μm film thickness, Agilent, Santa Clara, USA) connected by a modulator (G3486ACFT, Agilent, Santa Clara, CA, USA), to one segment of HP-INNOWAX (5 m × 0.250 mm i.d. × 0.15 μm film thickness, Agilent, Santa Clara, CA, USA). For the 2D column connected to the MS detector, a 0.50 m segment was used. A constant inlet pressure of 21.32 psi was selected to obtain a fixed split ratio between the two columns of about 1:20. The oven was programmed as 40 °C for 5 min, then gradually increased to 250 °C at 2 °C/min rate. Held for 15 min and finally raised to 270 °C at 10 °C/min. One μL of samples was injected at 250 °C automatically and in the splitless mode; Transfer line temperature, 270 °C.; Inlet pressure of carrier gas (He) was 21.32 psi (kept constant); Modulation delay 0.01 min; Modulation period 1.6 s; Inject time 0.15 s. MS conditions were as follows: Ionization voltage 70 eV; Transfer line temperature 280 °C; Interval scan: 35–300 *m*/*z*; Scan speed: 10,000 amu·s^−1^ (25 Hz). Helium was the carrier gas (1 mL/min). GC Image R2.4 GC×GC software was utilized for data acquisition and analysis. Identification of several compounds was carried out by comparing each compound mass spectra with in-house NIST 11, Wiley 9, and FFNSC 2 mass spectral database. The criterion for accepting a detected compound was a minimum of 90% similarity with the library. These identifications were also confirmed by other published mass spectra and linear retention indices (LRI). Linear retention indices were calculated using a series of *n*-alkanes (C_8_–C_40_). In addition, some of the compounds were confirmed by comparison of mass spectra and retention times with standard compounds.

### 3.3. Insects

Insect cultures were maintained at the Department of Agriculture Food and Forest Science at the University of Palermo (Italy) in the climatic room at 25 ± 2 °C, 50–60% r.h., and a 16:8 light:dark photoperiod. Different species were kept separated in plastic cages (25 × 25 × 40 cm) with two 5-cm diameter mesh-covered holes for ventilation. *Sitophilus oryzae* were fed with a mixture of wheat flour and rice 1:1 *w*/*w*; *L. serricorne* were fed with a mixture containing chamomile powder (100 g), 00 flour (95 g), 0 flour (60 g), bran (40 g), and brewer’s yeast (5 g); *R. dominica* colony was fed with whole wheat grains; *N. rufipes* colony was fed with a mixture of pet food enriched with dried fish and ham.

### 3.4. Toxicity Bioassays

Bioassays to evaluate the contact/fumigation toxicity of *C. maritima* and *L. siculum* EOs against *S. oryzae*, *L. serricorne*, *N. rufipes*, and *R. dominica* were carried out using 9-cm diameter glass Petri dishes. EOs were diluted in *n*-hexane (>99%, Sigma-Aldrich, Milan, Italy) at the concentration of 1%. An aliquot of 100 µL was gently pipetted in each internal face of the Petri dishes to cover all the surface. After 2 min for solvent evaporation, adult insects were released inside the Petri dishes with 2 g of food (same used for rearing). In consideration of the different adult sizes of the species tested and the availability of individuals of a similar age, for each replication, we used the following numbers of adults: twenty *S. oryzae*, twenty *L. serricorne*, eight *N. rufipes*, and ten *R. dominica*. Ten replications of each bioassay were carried out for each species and EO. As a control, an equal number of replications was carried out by pipetting 100 µL of *n*-hexane. After the start of the experiments, Petri dishes containing the individuals were transferred to the climatic room at 25 ± 2 °C and 16:8 L:D conditions. Toxicity was evaluated in terms of mortality by counting dead insects three days after the start of the experiment. Data obtained were analyzed using a one/way ANOVA, followed by Tukey’s test using STATISTICA 10.0 for Windows (Statsoft, Vigonza, PD, Italy).

## Figures and Tables

**Table 1:** Compounds identified in the essential oil of *Calendula incana* subsp. *maritima* using GC×GC-MS.

No	t^1^_R_ (min.s)	t^2^_R_ (min.s)	Compounds	LRI_exp_^a^	LRI_lit_^b^	Content (%) ^c^	Ident. ^d^
1	17.08	1.79	*β*-Pinene	977	981	*t*	1, 2, 3
2	17.24	1.63	2-Pentylfuran	991	993	*t*	1, 2
3	18.33	0.61	*p*-Cymene	1026	1028	*t*	1, 2, 3
4	18.60	1.72	Limonene	1031	1028	*t*	1, 2, 3
5	18.87	1.32	Benzeneacetaldehyde	1046	1045	0.08 ± 0.001	1, 2, 3
6	19.00	1.41	*β*-Terpinene	1048	1056	*t*	1, 2, 3
7	20.32	1.83	Nonanal	1106	1109	*t*	1, 2
8	25.11	1.86	Decanal	1206	1210	*t*	1, 2, 3
9	27.13	1.59	*α*-Cubebene	1359	1365	1.33 ± 0.02	1, 2
10	27.64	1.56	*α*-Copaene	1387	1381	0.12 ± 0.004	1, 2
11	27.88	1.63	*β*-Cubebene	1391	1397	1.15 ± 0.03	1, 2
12	28.85	1.53	*cis*-Muurola-3,5-diene	1432	1431	1.20 ± 0.02	1, 2
13	31.83	1.37	*γ*-Muurolene	1479	1480	0.39 ± 0.008	1, 2
14	32.04	1.03	4-*epi*-Cubebol	1491	1497	23.11 ± 0.74	1, 2
15	34.24	1.28	Cubebol	1518	1525	35.84 ± 0.69	1, 2
16	34.26	1.71	*δ*-Cadinene	1522	1523	0.71 ± 0.02	1, 2
17	34.32	1.34	*α*-Calacorene	1525	1526	0.80 ± 0.007	1, 2
18	35.21	0.89	Calamenene	1528	1531	1.02 ± 0.02	1, 2
19	35.91	1.13	Cadala-1(10),3,8-triene	1544	1552	1.43 ± 0.07	1, 2
20	36.23	0.81	Ledol	1549	1553	0.70 ± 0.010	1, 2, 3
21	40.75	1.27	Cubenol	1647	1648	12.59 ± 0.39	1, 2
22	40.84	1.03	*τ*-Muurolol	1651	1654	13.15 ± 0.44	1, 2
23	40.91	1.42	Ledene oxide-(II)	1653	1655	2.19 ± 0.08	1, 2
24	57.83	0.84	Hexahydrofarnesyl acetone	1842	1845	0.59 ± 0.010	1, 2
Monoterpene Hydrocarbons						*t*	
Sesquiterpene Hydrocarbons						8.150 ± 0.199	
Oxygenated Sesquiterpenes						88.17 ± 2.360	
Others						0.090 ± 0.002	
Total						96.410 ± 2.561	

t^1^_R_: first dimension retention time (minutes.seconds); t^2^_R_: second dimension retention time. ^a^ Linear retention index, obtained through the modulated chromatogram, reported for DB-5 MS apolar column; ^b^ Linear retention index reported for DB-5 MS column or equivalents reported in the literature; ^c^ Content is the peak volume percentage of compounds in the essential oil sample; ^d^: 1 = retention index identical to bibliography; 2 = identification based on comparison of MS; 3 = retention time identical to authentic compounds; *t*: traces, <0.05%. Compounds are classified in order of linear retention time of the apolar column.

**Table 2:** Compounds identified in the essential oil of *Laserpitium siler* subsp. *siculum* using GC×GC-MS.

No	t^1^_R_ (min.s)	t^2^_R_ (min.s)	Compounds	LRI_exp_^a^	LRI_lit_^b^	Content (%) ^c^	Ident. ^d^
1	12.59	0.68	3-Hexanone	782	775	*t*	1, 2
2	13.05	0.94	2-Hexanone	798	791	*t*	1, 2, 3
3	14.91	1.30	*cis*-4-Nonene	891	885	0.07 ± 0.002	1, 2
4	16.19	1.46	*α*-Pinene	937	936	*t*	1, 2, 3
5	16.72	1.56	Camphene	959	954	0.93 ± 0.010	1, 2, 3
6	17.02	1.77	*β*-Pinene	977	981	3.14 ± 0.15	1, 2, 3
7	17.29	1.67	2-Butyltetrahydro-furan	999	986	*t*	1, 2
8	17.50	1.72	2-Carene	1001	1003	2.07 ± 0.04	1, 2
9	17.62	1.51	*α*-Phellandrene	1003	1005	0.38 ± 0.010	1, 2, 3
10	17.91	1.15	Sylvestrene	1014	1021	0.11 ± 0.004	1, 2
11	17.96	1.62	3-Carene	1015	1010	0.23 ± 0.001	1, 2
12	18.23	1.82	*α*-Terpinene	1019	1017	3.57 ± 0.11	1, 2, 3
13	18.37	1.93	4-Carene	1022	1018	0.82 ± 0.010	1, 2
14	18.40	0.63	*o*-Cymene	1023	1022	0.55 ± 0.02	1, 2
15	18.56	1.72	*β*-Phellandrene	1029	1028	41.98 ± 0.93	1, 2, 3
16	18.60	1.72	Limonene	1031	1028	23.76 ± 0.78	1, 2, 3
17	19.00	1.41	*β*-Terpinene	1048	1056	11.83 ± 0.08	1, 2, 3
18	19.73	2.03	*α*-Terpinolene	1082	1087	0.73 ± 0.02	1, 2
19	19.99	1.62	2,4-Dimethylstyrene	1089	-	0.09 ± 0.003	1, 2
20	25.15	1.77	*α*-Terpineol	1207	1199	0.19 ± 0.002	1, 2, 3
21	26.54	2.14	4-Terpinenyl acetate	1291	1282	5.28 ± 0.24	1, 2
22	27.28	1.62	*β*-Bourbonene	1368	1385	*t*	1, 2
23	27.31	1.82	*α*-Ylangene	1369	1373	*t*	1, 2
24	27.64	1.56	*α*-Copaene	1387	1381	0.15 ± 0.003	1, 2
25	27.93	1.98	1,7-Dimethylnaphthalene	1396	1410	0.05 ± 0.001	1, 2
26	28.53	1.77	Caryophyllene	1425	1417	1.12 ± 0.03	1, 2, 3
27	28.61	1.98	*β*-Ylangene	1427	1422	0.59 ± 0.010	1, 2
28	28.87	1.51	*β*-Copaene	1433	1432	*t*	1, 2
29	29.45	1.62	*γ*-Elemene	1439	1434	0.21 ± 0.004	1, 2
30	30.01	1.98	Humulene	1455	1453	0.12 ± 0.002	1, 2
31	31.17	2.03	Patchoulene	1468	1467	0.44 ± 0.007	1, 2
32	31.79	0.63	*α*-Curcumene	1478	1483	0.06 ± 0.001	1, 2
33	33.66	0.21	*α*-Bulnesene	1511	1506	0.09 ± 0.006	1, 2
34	34.21	0.31	*δ*-Cadinene	1519	1522	0.16 ± 0.009	1, 2
35	35.43	0.99	*β*-Vatirenene	1536	1542	0.09 ± 0.006	1, 2
36	42.67	1.88	*trans*-Nuciferol	1698	-	0.19 ± 0.008	1, 2
37	48.07	1.93	*β*-Nootkatol	1725	1712	0.05 ± 0.002	1, 2
38	51.15	0.63	Chamazulene	1731	1730	0.13 ± 0.005	1, 2
Monoterpene Hydrocarbons						90.19 ± 2.168	
Oxygenated Monoterpenes						5.47 ± 0.242	
Sesquiterpene Hydrocarbons						3.16 ± 0.083	
Oxygenated Sesquiterpenes						0.24 ± 0.010	
Others						0.13 ± 0.004	
Total						99.19 ± 2.507	

t^1^_R_: first dimension retention time (minutes.seconds); t^2^_R_: second dimension retention time. ^a^ Linear retention index, obtained through the modulated chromatogram, reported for DB-5 MS apolar column; ^b^ Linear retention index reported for DB-5 MS column or equivalents reported in the literature; ^c^ Content is the peak volume percentage of compounds in the essential oil sample; ^d^: 1 = retention index identical to bibliography; 2 = identification based on comparison of MS; 3 = retention time identical to authentic compounds; *t*: traces, < 0.05%. Compounds are classified in order of linear retention time of the apolar column.

## Data Availability

The data presented in this study are available on request from the corresponding authors.

## Sample Availability

Samples of the compounds of *C. maritima* and *L. siculum* are available from the authors.
